# Engagement With a Relaxation-Based Mobile Health Intervention for Perioperative Anxiety: Prospective Longitudinal Cohort Mixed Methods Study

**DOI:** 10.2196/64278

**Published:** 2026-09-18

**Authors:** Xinghui Yan, Afton L Hassett, Jennifer F Waljee, Mark W Newman, Sun Young Park, Rongqi Bei, Noelle E Carlozzi

**Affiliations:** 1 School of Information University of Michigan Ann Arbor, MI United States; 2 Chronic Pain & Fatigue Research Center Department of Anesthesiology University of Michigan Ann Arbor, MI United States; 3 Department of Surgery Michigan Medicine Ann Arbor, MI United States; 4 Department of Surgery Indiana University Indianapolis, IN United States; 5 Electrical Engineering and Computer Science College of Engineering University of Michigan Ann Arbor, MI United States; 6 Penny W Stamps School of Art and Design University of Michigan Ann Arbor, MI United States; 7 Center for Clinical Outcomes Development and Application (CODA) Departments of Physical Medicine and Rehabilitation, and Surgery University of Michigan Ann Arbor, MI United States

**Keywords:** mHealth intervention, perioperative anxiety, pain management, user engagement, relaxation, mindfulness

## Abstract

**Background:**

Relaxation-based mobile health (mHealth) interventions hold strong potential to address perioperative anxiety and pain management in a scalable manner. However, there is limited research on how surgical patients engage with these interventions and what aspects may be most helpful.

**Objective:**

This study had 3 goals: (1) explore how users interact with a relaxation-based mHealth intervention during the perioperative period; (2) understand how user performance and perceptions relate to perceived benefits; and (3) identify design considerations to enhance user engagement and behavior change outcomes in mHealth interventions.

**Methods:**

We conducted a prospective longitudinal cohort, mixed methods study to evaluate user engagement with MiCarePath, a relaxation-based mHealth intervention. A total of 19 perioperative patients (mean age 41.3 years; 14/19, 74%, female) undergoing elective surgery and reporting sometimes-to-always anxiety were enrolled using 3 recruitment methods. Participants used the app from 10 days before surgery to 4 weeks after surgery. We collected quantitative data on video engagement metrics (eg, duration of watching, response rate to video prompts) and ecological momentary assessments of anxiety and pain. Concurrently, we conducted semistructured, data-prompted interviews at 3 time points to assess user perceptions. These data were integrated using descriptive statistics, repeated-measures ANOVA, and qualitative thematic analysis to examine the relationship between user performance and perceived benefits.

**Results:**

Eleven participants reported developing or improving relaxation-based self-management strategies (high benefit), whereas 8 reported little benefit from the intervention. The high-benefit group showed significantly greater total watching time (difference 223.8 minutes, 95% CI 95.54-366.46 minutes, *P*<.001), higher adherence to video prompts (73.45% vs 34.60%, *P*<.001), and greater perceived helpfulness of the content (79.80% vs 25.50%, *P*<.001) compared with the low-benefit group. A significant group-by-time interaction effect was observed for engagement (*F*_1,17_=17.02, *P*<.001). No difference was observed in delay in response to video prompts. However, within each group, participant perceptions were not fully reflected in performance. Some participants in the high-benefit group showed decreased video watching after surgery but still reported feeling engaged with the intervention. Interview data revealed that “intentional use” behavior—where users actively seek content to manage symptoms independent of prompts—distinguished these participants. Those who developed intentional use were able to practice relaxation techniques without the app and resume app engagement following contextual disruptions.

**Conclusions:**

This study explored user engagement with a relaxation-based mHealth intervention for perioperative care, advancing the literature by integrating user perceptions and performance for digital anxiety and pain management. Results indicate that perceived benefit may not always be reflected in user performance. Intentional use emerges as a promising indicator of effective mHealth engagement in perioperative care. Future designs should aim to foster intentional use through features such as reflection prompts and adaptive notifications. Future work should also develop computational models to detect intentional use and evaluate adaptive interventions in larger cohorts.

## Introduction

### Background

Preoperative anxiety is common and closely intertwined with pain experiences both before and after surgery. It affects a significant proportion of individuals across different ages and health conditions, with estimates suggesting that up to 50% of patients experience heightened anxiety in anticipation of surgery [1]. Some studies report even higher rates, with up to 80% of patients across different surgical fields experiencing anxiety due to elevated stress and fear of surgery [2]. The experience of preoperative anxiety, if not addressed in a timely and appropriate manner, often extends throughout the entire perioperative period. Greater preoperative anxiety is associated with adverse outcomes, including increased patient-reported pain, prolonged opioid use, and postoperative misuse [3-6]. These interrelated challenges pose a substantial clinical burden, increasing both clinical workload and patient morbidity [7]. Despite the severity of the problem, managing both anxiety and pain throughout the perioperative period remains a critical challenge. Unlike general chronic disease populations, perioperative patients often face unique, acute stressors that complicate self-management. These include physical mobility restrictions, the cognitive load of managing new medication regimens (eg, opioids), and the emotional burden of anticipated surgical outcomes, all of which significantly complicate recovery for patients undergoing surgery [3,8].

In recent years, mobile health (mHealth) interventions have been shown to promote well-being through personalized care and behavioral support [9-11] and have the potential to improve perioperative outcomes by delivering interventions that support healthy behaviors [10,12]. With integrated educational and psychological strategies delivered via an mHealth platform, patients can receive support without the need for additional clinical appointments or programs [13,14], allowing more time to build resilience and receive behavioral support before and after surgery [15]. Some interventions focus on behavioral pain management and self-regulation, helping patients actively participate in their recovery and pain control [16,17]. A growing number of intervention apps have adopted a relaxation-based approach, leveraging evidence-based mind-body techniques to alleviate perioperative anxiety and pain [18]. These include guided mindfulness, breathing exercises, meditation [19], progressive muscle relaxation [20], and music-based interventions delivered via digital devices [21]. Emerging evidence suggests that these technologies show promise in supporting patients’ emotional and physical well-being in a scalable manner during the perioperative period.

### Research Gap

While mHealth interventions show promise for managing preoperative anxiety and pain, sustained user engagement remains a major challenge in this and most mHealth settings [22-26]. User engagement refers to interaction with and use of mHealth tools and can be measured by user perceptions and performance [22,23]. The effectiveness and desired outcomes of mHealth interventions are often hindered by poor uptake [27], early attrition [28], significant declines in adherence over 4-8 weeks of an intervention [29], intentional nonadherence to the intervention [30], and even abandonment [23] (eg, [29,31,32]). While broader mHealth studies have examined engagement, there is a scarcity of studies examining both user performance and perceptions in relaxation-based interventions for surgical patients [15,20,33]. Prior work often focused on general populations or chronic conditions, lacking the specific context of the acute perioperative phase and longitudinal data tracking usage from presurgery to recovery. Consequently, the intersection of objective usage metrics and subjective user experience remains underexplored in the perioperative patient population.

Given the acute stress of surgical anticipation, significant time constraints, and the physical burden of postoperative pain, these patients often navigate competing medical priorities that may limit their capacity to engage with digital tools [34,35]. Consequently, prior research on digital interventions for pain and anxiety management in surgical settings has reported low uptake and high attrition due to substantial time demands and emotional burden [16,34]. However, existing studies have relied heavily on quantitative metrics (eg, system usage data) to assess these challenges, often neglecting the affective and cognitive dimensions of the user experience [35]. Taken together, only a few studies have examined both user performance and perceptions through a longitudinal lens specifically within the acute perioperative phase, as most research focuses on general wellness or chronic conditions. This gap is particularly significant for perioperative patients, whose unique stressors and needs may shape their interaction with mHealth interventions for anxiety and pain management. Thus, a comprehensive understanding of the relationship between objective usage and subjective experience remains underexplored in this population.

To address this gap, we investigated perioperative patient engagement with a relaxation-based mHealth intervention, MiCarePath. MiCarePath was designed to provide perioperative patients with low-cost, engaging education and relaxation-based exercises to help reduce both anxiety and pain. It includes 3 short educational videos about surgery in general (eg, what to expect, pain as a normal part of healing), the role of anxiety in the experience of pain, the use of opioids and other strategies to mitigate surgical pain, and the potential benefits of learning behavioral skills to reduce anxiety and pain. MiCarePath also includes self-directed, evidence-based relaxation exercises, such as guided imagery meditation videos and deep breathing training, which have been demonstrated to reduce perioperative anxiety and pain [3,4]. In addition, MiCarePath incorporates ecological momentary assessments of anxiety and pain, as well as questionnaires to measure health and behavioral outcomes (eg, pain, anxiety, self-management strategies) at different time points during the study.

Regarding user performance, we primarily focused on the length of interaction with different videos, the delay in responding to video prompts, and the response rate to video prompts. To examine user perceptions, we conducted semistructured interviews informed by the data-prompted interview approach [36] both before and following surgery. We used video engagement data, momentary anxiety and pain levels, and feedback related to the relaxation videos to probe participant decision-making and actions in response to video prompts. Findings from this study will inform the design of relaxation-based interventions for perioperative anxiety and pain management and enhance understanding of user perceptions and performance with such interventions.

### Objective

We aimed to better understand perioperative patients’ engagement with a relaxation-based mHealth intervention. We examined both user perceptions and performance during the pre- and postsurgical periods. This understanding is critical for designing more effective, patient-centered interventions that promote adherence and improve clinical outcomes. We conducted a mixed methods analysis to address the following research questions: (1) How do patients act on video prompts and engage with relaxation exercise videos during the perioperative period? (2) How can user perceptions and performance be aligned with an intervention to better understand user engagement? and (3) How might mHealth interventions be better designed to enhance user engagement and ultimately improve behavioral outcomes?

## Methods

### Intervention Design: The MiCarePath App

The MiCarePath app was designed to help surgical patients manage perioperative anxiety and pain through education and the teaching of relaxation skills. The app was built using MyDataHelps [37], a research platform that enables survey data collection and the delivery of mHealth interventions. The MiCarePath intervention included 5 components: (1) 3 surgery-related educational videos assigned to participants at the start of the intervention, covering expectations for surgery, safe opioid use, and the benefits of learning anxiety and pain self-management strategies; (2) relaxation exercise videos for day-to-day pain and anxiety management, including guided imagery meditation videos and a deep breathing pacer; (3) push notifications that provide reminders to watch relaxation exercise videos; (4) mindfulness-related behavioral suggestions; and (5) ecological momentary assessments of anxiety and pain (1 item each).

Prior literature shows that the timing of intervention delivery affects user receptivity and engagement with intervention content [12,25,26]. While we did not aim to evaluate the effectiveness of the intervention in a fully randomized controlled trial setting, we recognized the importance of randomizing the timing of video prompts to examine user experience. This approach allowed us to explore context-specific variations in user decision-making and actions in response to different video prompts. Accordingly, each day, the app delivered a video prompt at a random time within 1 of the following 4 time frames: (1) morning: 9:00 AM-noon; (2) early afternoon: noon-3:00 PM; (3) late afternoon: 3:00-6:00 PM; and (4) evening: 6:00-9:00 PM. Similar to the video prompts, participants completed momentary assessments of pain and anxiety 4 times per day, prompted within the same time frames. Considering the capabilities of MyDataHelps, we randomly generated 3 unique prompting schedules (ie, when to deliver a relaxation exercise video and ecological momentary assessments) and randomly assigned participants to one of these schedules, forming 3 groups. Participants within the same group received intervention prompts and self-report questions at the same time.

The primary intervention content of the MiCarePath app was the relaxation exercise videos, which were presented to participants once per day and were also the focus of this study. The videos, ranging from approximately 10 to 15 minutes in length, immerse users in various relaxation and meditation scenarios, such as a forest, seaside, or garden. These videos were developed and produced specifically for the MiCarePath app. Participants could switch to a different video or watch multiple videos, and they also had the option to proactively watch a video without being prompted. After completing relaxation exercises, the app prompted them to answer 3 or 4 questions designed to collect user feedback, including whether the video was watched, the perceived helpfulness of the video (helpful, neutral, or not helpful), activities before responding to the prompt, and reasons for nonadherence (if relevant). To monitor users’ anxiety and pain levels, we implemented ecological momentary assessments using two 10-point single-item rating scales (ie, “From 1-10, what is your level of anxiety/pain right now?”) [37].

To preliminarily assess the major outcomes of the MiCarePath intervention, the app collected data on participants’ pain, anxiety, and any self-management strategies used over the course of the intervention. These measures were completed at study entry and for 2 weeks following surgery. The small sample size precluded analysis of the intervention’s effectiveness; instead, we examined participants’ user experience over time to explore intervention engagement.

### Study Procedures

The study team first invited individuals to participate. The study used a fully remote design, with participant coordination conducted via email. Individuals who indicated interest were screened for eligibility criteria, consented, and then provided with instructions to download and install the MiCarePath project within the MyDataHelps app. The intervention period for each participant lasted approximately 40 days; participants began using the app at least 10 days before surgery and were encouraged to continue for 1 month following surgery. This timeline allowed participants to become familiar with the intervention, as well as to learn and practice relaxation techniques in preparation for surgery and during recovery. We conducted 3 qualitative interviews (each approximately 15-20 minutes): (1) before surgery (1-2 days prior); (2) 2 weeks after surgery; and (3) upon study completion (4 weeks following surgery, approximately 30 minutes). These interviews, conducted at multiple time points over the course of the intervention, allowed us to examine changes in participant perceptions and experiences. Figure 1 presents the study timeline. Additionally, participants were asked to complete questionnaires at these time points, including measures of anxiety and pain levels, self-management strategies, and medication use.

Interviews were informed by a data-prompted interview approach, which helps researchers gain rich insights into participants’ in-the-field experiences by using collected data to guide conversations [38-40]. Two authors (XY and RB), both graduate researchers trained in human-computer interaction, developed the interview guide and conducted the interviews remotely via Zoom (Zoom Communications). During each interview, the research investigator first introduced the purpose of the interview and their background, and began with general questions about health and surgical status. We then presented data visualizations to participants displaying information about their actions in response to video prompts (ie, whether they chose to watch the video or not, or switched to a different video), video engagement (eg, completion of a video), momentary anxiety and pain, and activities before watching a video. Interview materials were presented using Google Slides (Google LLC; see Figure 2 for an example). Specifically, we probed user actions when (1) the participant chose not to watch a video; (2) the participant appeared to watch a video at an atypical time or in a unique context; and (3) the participant expressed an attitude that differed from their usual perceptions of the videos. When presenting the visualizations, the researcher described the data and asked participants to recall their experiences around the time they responded to a video prompt. Follow-up questions were used to explore possible reasons underlying participant decision-making and actions. We concluded the interviews by asking participants about other techniques or behavioral support they received during that period.

**Figure 1:**
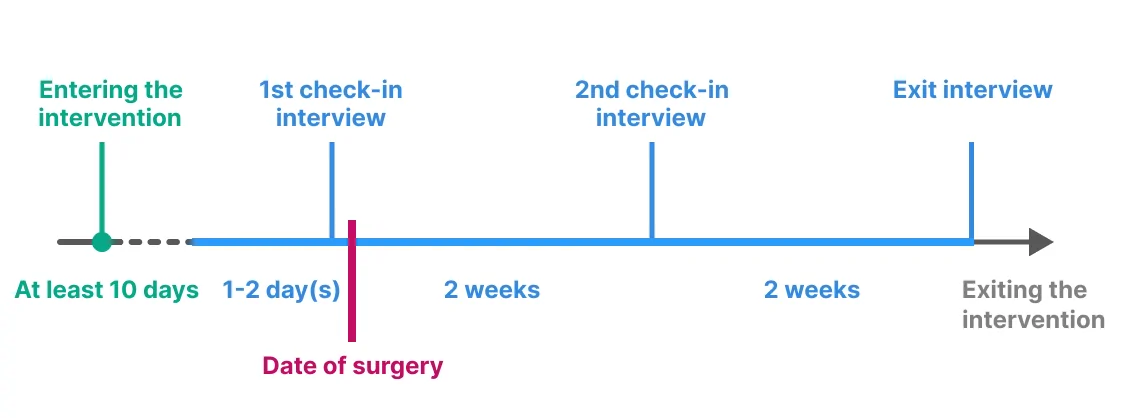
Study timeline. Participants attended 2 check-in interviews and 1 exit interview.

**Figure 2:**
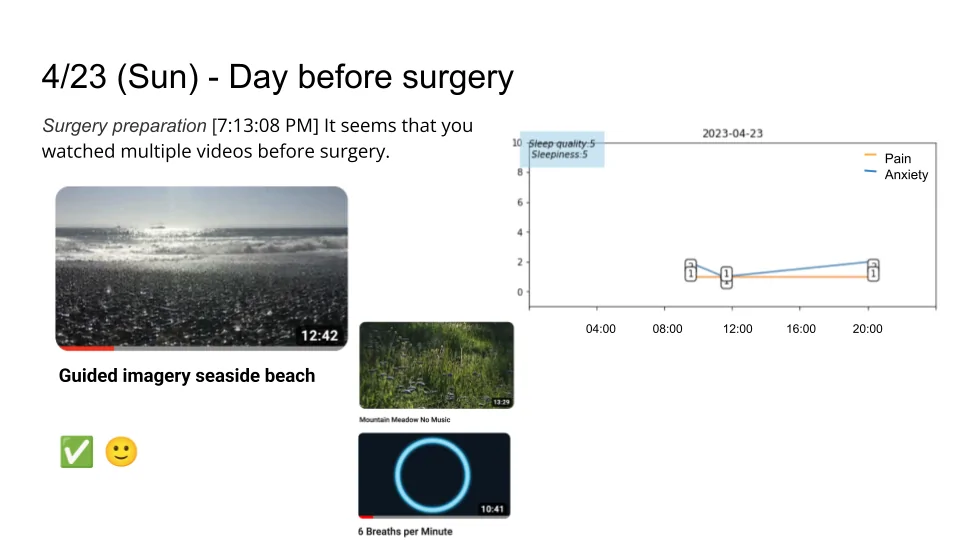
Example of the visualization shown during interviews. Text indicated when participants received a video prompt and their subsequent actions (eg, watching the video). A video screenshot was used to prompt recall of past experiences, and same-day pain and anxiety scores were displayed. Icons indicated video completion and perceived helpfulness.

### Participant Recruitment

Participants were recruited using 3 sampling methods: study posts on a university-based research study site, snowball sampling of clinical providers’ patient contacts, and review of surgical schedules for patients undergoing elective surgery. We used multiple recruitment methods to maximize enrollment of a diverse cohort of end users. Eligible participants were (1) at least 18 years of age; (2) fluent in English; (3) undergoing elective surgery at least 10 days after the consent date (to provide sufficient time to engage with the app); (4) willing to participate in surveys through 4 weeks after surgery; (5) able to access a device (smartphone or tablet, such as an iPad; Apple Inc) to use the MyDataHelps platform for completing anxiety and pain assessments and engaging with the MiCarePath intervention content; and (6) endorsing surgery-related anxiety, as indicated by a response of 3-5 to the following statement: “I feel anxious when thinking of my upcoming surgery (1=never, 2=rarely, 3=sometimes, 4=often, and 5=always).” We focused on patients who reported surgery-related anxiety, as these patients may be more interested in learning relaxation techniques and motivated to do so before surgery. The enrollment process commenced in October 2022 and concluded in July 2023. We aimed to enroll 15-25 participants to achieve thematic saturation and allow for subgroup comparisons [41,42]. The final sample size (N=19) is consistent with prior mixed methods and feasibility studies examining mHealth engagement in clinical contexts [29,43]. Given the exploratory aim of identifying engagement patterns and intentional use behaviors, this sample size was deemed sufficient to support thematic analysis and descriptive quantitative comparisons rather than hypothesis testing.

### Ethical Considerations

Ethics approval was obtained in 2022 from the University of Michigan, Ann Arbor Institutional Review Board (approval number IRBMED HUM00217162). Individuals who contacted the study team were screened for eligibility, and those who were eligible provided e-consent before enrollment in study activities. Participants were instructed to install the MyDataHelps app during a brief onboarding session and were provided with a 1-page user manual for reference.

To ensure participant data confidentiality, all collected data were deidentified and replaced with unique participant codes. Interviews were audio-recorded, and no images of participants were obtained or reported in the manuscript. The document linking these codes to personally identifiable information was stored separately from the study data on a secure, firewalled university server, with access strictly restricted to the research team. Regarding data security, the MyDataHelps platform uses industry-standard encryption protocols. It is built on the HIPAA (Health Insurance Portability and Accountability Act)–compliant HIEBus platform, which securely handles protected health information in accordance with clinical standards.

Participants were compensated up to US $60 for completing surveys and interviews. They were not compensated for their engagement with video prompts to mitigate potential intervention effects related to incentives.

### Data Analysis

#### Characterization of Perceived Benefit and Mixed Methods Integration

As the key aim of the MiCarePath intervention is to introduce relaxation techniques to perioperative patients and encourage them to adopt or maintain relaxation as a self-management strategy, we used participant-reported use of strategies at week 2 after surgery—a multiple-choice question framed as follows: “Do you currently use strategies to decrease anxiety? (check all that apply).”—and frequency of use as criteria to assess perceived benefit from the intervention (ie, whether patients learned and developed a new self-management strategy as intended). We categorized participants into 2 groups based on their reported benefit from the intervention: a high-benefit group, comprising those who reported developing a new self-management strategy or maintaining relaxation techniques, and a low-benefit group, comprising those who did not report these outcomes. Our focus was on identifying commonalities within each perceived benefit group (high vs low) and differences between the 2 groups to identify phenomena indicative of users’ perceived benefit. We first followed a sequential explanatory strategy [43,44], using the collected data to inform the check-in and exit interviews. During the data analysis phase, researchers applied a concurrent triangulation approach to conduct qualitative and quantitative analyses simultaneously and then integrated both sets of results for interpretation. When discrepancies were identified, we used participants’ interview data to interpret changes in perceptions and performance.

#### Quantitative Data Analysis

We analyzed 4 key video engagement metrics: (1) average length of video engagement per week (in minutes), (2) response rate to video prompts (defined as whether a participant opened the notification), (3) response delay (measured in seconds from notification to video engagement), and (4) reported helpfulness of videos (categorized as helpful, neutral, or not helpful). These measures served as proxy indicators of user interest and depth of engagement with different relaxation videos [45]. User interaction data were collected using Google Analytics 4 and Google Tag Manager [46], which recorded time-stamped events such as video start and stop times, video content type, and engagement duration. Weekly engagement data (eg, postoperative week 1, week 2) were treated as a categorical within-participant variable to enable temporal comparisons. Missing data resulting from participant dropout were handled using listwise deletion, as analyses focused on within-participant trends rather than population-level inference. Given the exploratory nature of the study and small sample size, analyses were interpreted descriptively rather than as confirmatory tests. We began with descriptive statistical analyses to summarize trends in video engagement and perceived helpfulness. We then applied mixed-design analysis of variance (mixed ANOVA) to examine weekly video viewing time and response delay between the 2 groups. Before conducting ANOVA, we assessed the assumptions of normality and sphericity. When the Mauchly test indicated a violation of sphericity, we applied the Greenhouse-Geisser correction. Given the exploratory nature of the study, we did not apply corrections for multiple comparisons. All statistical analyses were conducted in Python (version 3.8.7; Python Foundation) using Jupyter Notebook.

#### Qualitative Data Analysis

We conducted a 2-stage thematic analysis of interview data, drawing on data-prompted interview analysis practices [38-40]. Interviews were audio-recorded and transcribed using auto-generated transcripts, which were subsequently proofread by the first author (XY) for accuracy. Researchers first reviewed participants’ transcripts and annotated critical incidents, a widely used technique in qualitative research in which researchers focus on participants’ recalled events (eg, how they responded to an intervention prompt) to reconstruct past behaviors and identify notable behavioral patterns [47]. Data visualizations presented during the interviews (eg, engagement trends) were used to contextualize participant responses and anchor incidents during analysis. In the first stage, 2 researchers (XY and RB) engaged in memo writing [48] to summarize each participant’s key characteristics (including prior meditation experience and coping strategies for anxiety and pain management) and general experience with the intervention (including development of new self-management strategies and overall attitudes toward the intervention). Using a spreadsheet, researchers conducted a deductive analysis guided by the study’s research questions, focusing on key experiential domains such as perceptions, reported behaviors, and changes before and after surgery. The spreadsheet format allowed for flexible categorization, efficient organization, and filtering of quotes by contextual variables (eg, timing relative to surgery, user group). Drawing on participants’ objective engagement data, the researchers examined alignment between perceptions and performance, particularly across the 2 user groups. Check-in interview data were used to help explain changes in engagement over time. This triangulation process allowed us to contextualize subjective experience with objective intervention engagement patterns, specifically examining alignment between user perceptions and performance across the 2 user groups.

Researchers then applied in vivo coding [49,50] to uncover emergent themes grounded in participants’ language through an iterative codebook development process. They collaboratively coded around 20% of the transcripts (4/19, 21%), establishing intercoder reliability through discussion and alignment on code definitions. Once a consistent agreement was reached, the remaining transcripts were independently coded using the finalized codebook. The broader research team, including senior members (NEC and MWN), held regular debriefing meetings to review and refine emerging themes. Data saturation was assessed through ongoing review of new transcripts and was considered achieved when no substantial new codes or themes emerged.

To ensure the quality and transparency of our mixed methods approach, this study followed the GRAMMS (Good Reporting of a Mixed Methods Study; Multimedia Appendix 1) framework [51] and the COREQ (Consolidated Criteria for Reporting Qualitative Research; Multimedia Appendix 2) guidelines [52].

## Results

### Overview

A total of 22 participants were enrolled in this study. Of these, 19 participants successfully completed the study; 1 participant dropped out before surgery, and 2 dropped out after surgery. Sixteen participants completed all 3 interviews, while 3 were either unavailable due to physical therapy or remained unresponsive to the research team’s phone calls or emails. Table 1 presents demographic characteristics, with a focus on prior experience and whether participants developed or maintained relaxation as a self-management strategy upon completing the intervention, which was used as the criterion to differentiate users’ perceived benefit.

Based on participant responses to the self-management strategy survey items and the reported frequency of use in the baseline and exit surveys, participants were categorized into 2 groups: a high-benefit group (n=11) and a low-benefit group (n=8). In the high-benefit group, 7 participants incorporated relaxation as a new self-management strategy, and 4 reported increasing the frequency of its use. By contrast, participants in the low-benefit group reported that they did not develop a new strategy.

**Table 1:** Demographic characteristics of the participants (N=19) who completed this study.

Characteristics			Perioperative patients, n
**Age group (years)**			
	20-34	6	
	35-49	4	
	50-64	5	
	65-80	4	
**Gender**			
	Male	4	
	Female	14	
	Chose not to disclose	1	
**Prior experience with relaxation exercises**			
	Yes	5	
	No	14	
**Self-management strategy**			
	Developed a new self-management strategy	7	
	Increasingly applied relaxation due to the intervention	4	
	Did not develop a new strategy	8	

### Comparing User Perceptions and Performance Between High- and Low-Benefit Groups

#### User Performance

All participants viewed the 3 educational videos upon entering the intervention. Table 2 presents the average total watching time, response rate to video prompts, delay in responding to a video prompt, and perceived helpfulness of the videos. Overall, results showed that, except for the average delay in responding to a video prompt, participants in the 2 groups exhibited significant differences in average total watching time (*P* <.001) and average response rate to video prompts (*P*<.001; also see Tables 2 and 3; Figures 3 and 4). The average weekly watching time and CIs of both high- and low-perceived benefit groups are presented in Table 3.

Before surgery, participants in the high-benefit group generally adhered to video prompts, with a mean nonadherence rate of only 11% (14/127). Four participants reported intentionally choosing their preferred relaxation exercises at least once, despite being recommended a different one. Three participants from the high-benefit group reported that they sometimes watched more than 1 video when prompted. In comparison, participants who perceived little or low benefit initially tried relaxation exercises before surgery, with an adherence rate of 55% (53/97) to video prompts, but most did not finish viewing the entire video. After surgery, 4 of 8 participants in the low-benefit group rarely attended to any video prompts.

A mixed ANOVA was conducted to examine changes in watching time across 5 perioperative time points (T1: before surgery; T2-T5: week 1 to week 4 after surgery) between the 2 groups. The data showed that, in the high-benefit group, the average weekly watching time started at 99.6 (95% CI 70.9-128.2) minutes at baseline (T1) and was maintained at 55.4 (95% CI 31.7-79.1) minutes by the end of the study (week 4, T5). Although there was a nominal reduction of 44.2 minutes, high interparticipant variability across the 5 time points resulted in a nonsignificant main effect of time (*P*=.10).

By contrast, participants in the low-benefit group exhibited a significant decline in watching time after surgery (*F*_4,28_=19.76, *P*<.001). While they began with moderate engagement at baseline (mean 47.0 minutes; 95% CI 25.6-68.4 minutes)—approximately half the watching time of the high-benefit group, the average weekly watching time in the low-benefit group had dropped to only 4.3 (95% CI 0.0-12.6) minutes. At baseline (T1), there was already a significant difference in watching time between the high- and low-benefit groups (*F*_1,17_=21.62, *P*<.001), suggesting that participants in the low-benefit group were not initially engaged with the intervention. Moreover, preliminary analyses indicated an interaction effect between perioperative time and participants’ perceived benefit level. The high-benefit group sustained 55.6% (55.4/99.6) of their baseline engagement by week 4 (maintaining a mean of 55.4 minutes), whereas the low-benefit group exhibited a significantly greater decline in watching time over time than the high-benefit group (*F*_1,17_=17.02, *P*<.001). Conversely, no significant difference in video responsiveness was observed between the 2 groups (*P*=.81). The average delay in attending to video prompts was comparable between high-benefit (5.54 hours) and low-benefit (5.15 hours) participants.

**Table 2:** The average total watching time, average response rate to video prompts, average delay in attending to a video prompt, and the rate of perceiving videos as helpful from the ecological momentary assessment. Except for the delay in attending to a video prompt, participants in the high-benefit group acted significantly more engaged with the intervention as opposed to the low-benefit group.

Group variable	High-perceived benefit (H) (n=11), mean (SD); 95% CI	Low-perceived benefit (n=8), mean (SD); 95% CI	*P* value
Total watching time on average (minutes)	340.8 (152.3); 238.5-443.1	117 (115.5); 20.4-213.6	<.001
Average response rate to video prompts (%)	73.45 (15.36); 63.13-83.78	34.60 (18.26); 19.35-49.90	<.001
Average delay in attending a video prompt (hours)	5.54 (4.02); 2.84-8.24	5.15 (3.42); 2.29-8.01	.81
Rate of perceiving videos as helpful (%)	79.80 (9.25); 73.18-86.42	25.50 (14.76); 13.20-37.80	<.001

**Figure 3:**
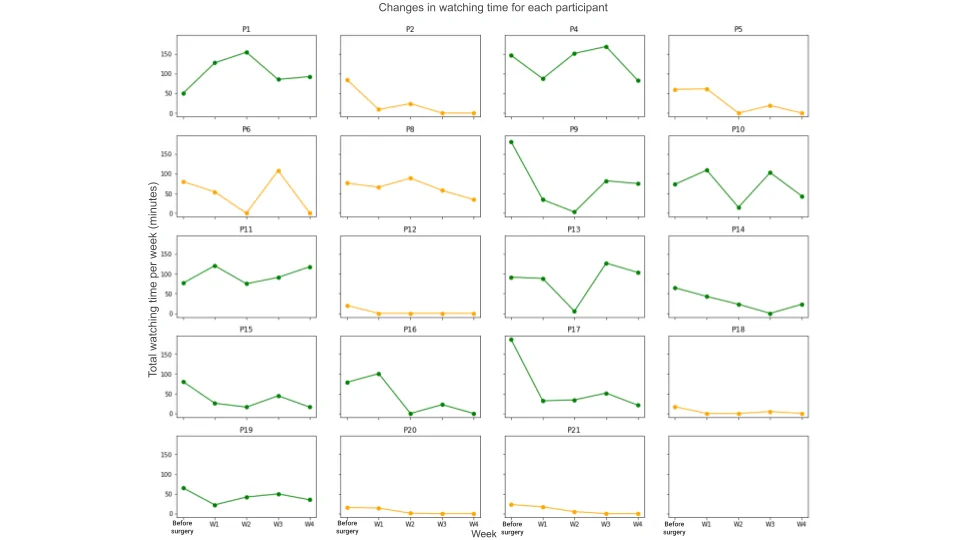
Weekly video engagement time for each participant relative to surgery (before surgery and weeks 1-4 after surgery). Participants in the high-benefit group are shown in green and those in the low-benefit group in yellow.

**Figure 4:**
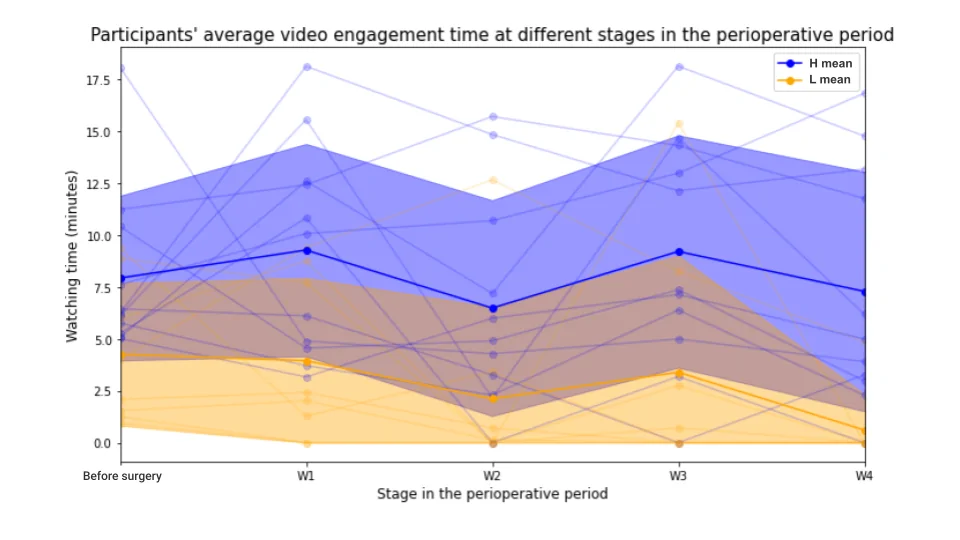
Participants’ average daily video engagement (minutes watched) over the intervention period, from enrollment to up to 4 weeks after surgery. For each group, the figure shows individual trajectories, the group mean (bold lines), and within-group variability (shaded areas).

**Table 3:** The average weekly watching time and CIs of both high- and low-perceived benefit groups.

Time point	High-perceived benefit (n=11), mean; 95% CI	Low-perceived benefit (n=8), mean; 95% CI
T1 (before surgery)	99.6; 70.9-128.2	47.0; 25.6-68.4
T2 (week 1)	71.8; 47.8-95.9	25.8; 5.6-46.1
T3 (week 2)	48.7; 15.9-81.5	14.3; 0.0-35.9
T4 (week 3)	65.3; 39.5-91.2	23.2; 0.0-50.7
T5 (week 4)	55.4; 31.7-79.1	4.3; 0.0-12.6

#### User Perceptions

##### High-Benefit Group (n=11)

All participants in the high-benefit group reported incorporating relaxation practices as a new self-management strategy or reinforcing their previous experience with relaxation. Participants with prior experience continued to engage with the videos, viewing at least one video per week during the intervention. For example, P4 had over 20 years of meditation experience and told us, “The meditation videos in this app were good because they were new to me. So it gave me a fresher feeling.” Among participants without prior experience (7/11), individuals gradually became familiar with relaxation techniques and appreciated having a tool to cope with anxiety and pain. Their positive experience with the intervention appeared to stem from 1 or more helpful relaxation exercises. For example, P9 tried garden imagery meditation for the first time and appreciated that the video offered less instruction and greater flexibility for imagination: “There's a little bit less instruction. The video does not talk as much, but it will give good ideas of going through the (garden) path and everything, and then just sitting down and relaxing. There's a little bit of freedom just to do whatever. And sometimes I like to imagine lots of flowers, or sometimes I just relax, and it's nice.” Similarly, P11 gradually became more engaged with the relaxation videos, and increased interaction reinforced continued use of the intervention: “I did some meditation sometimes, but on and off. But with this [intervention], it’s helping me to remind me to keep meditating and relaxing.”

##### Low-Benefit Group (n=8)

As noted in user performance, participants who perceived low benefit were not engaged with the intervention from the outset. Most participants in this group reported that they felt it was less necessary to incorporate the intervention into their self-care, owing to satisfaction with existing self-management strategies. For example, P6 reported being satisfied with his current strategy: “As long as I am just sitting in this massage chair, it’s not too painful, and I just watch television or read....This is the first time I really ever even thought about it [meditation] or seen anything about it.” For several participants, although they completed the educational videos (including the rationale for learning relaxation techniques) upon entering the intervention, they remained unreceptive to relaxation exercises and intentionally ignored video prompts, as reported by P5: “I’m also very busy with work, and I don’t have time [to use the intervention app]. If I have time, I’ll be relaxing and thinking about the surgery, and then I’ll get the notification. And I have to think about it again. It’s not that stressful, but sometimes I feel like I have one more thing to do now.”

### Discrepancy Between Participants’ Performance and Perceptions

As previously noted, for most participants, those who derived the most benefit from the intervention exhibited significantly higher quantity and quality of video engagement with the MiCarePath app (ie, average total watching time, *P*<.001; average response rate to video prompts, *P*<.001; and rate of perceiving videos as helpful, *P*<.001). However, when aligning each participant’s performance data with their perceptions of the app obtained from interviews, we observed variability within both groups. Briefly, a few participants in the high-benefit group showed lower video engagement at certain points during the intervention than some participants who reported little benefit. Additionally, although these participants appeared to perceive benefit from the MiCarePath app, their video engagement fluctuated throughout the intervention and occasionally showed signs of disengagement (eg, P14, P20, P21; see Figure 3). Conversely, 2 participants in the low-benefit group exhibited higher adherence rates before surgery, despite reporting that they did not perceive any benefit from using the intervention.

### High-Perceived Benefit With Low Intervention Engagement After Surgery

Five participants in the high-benefit group reported reduced use of the intervention after surgery, while others reported maintaining a similar level of use as before; this was also confirmed by their performance data. However, these 5 participants still reported that the videos were helpful and that they derived benefit from the relaxation exercises. Notably, all 5 participants refrained from using the intervention for at least one week during the postoperative period (1 participant did not use the intervention in week 1 after surgery, and 4 in week 2 after surgery). From the interviews, one possible explanation was that these participants were prescribed other treatments or therapy after surgery and therefore, felt less able to attend to the video prompts. Nevertheless, they expressed satisfaction whenever they engaged with a relaxation video. In P17’s case, he was proactively engaged with the videos before surgery to cope with abrupt, severe back pain, resulting in an average of 18 minutes of watching time per day: “That [the back pain] was a big thing. I was dealing with the pain and the anxiety that it might affect my heart surgery.” He reported a positive experience with the app: “For most of the videos, I ended up closing my eyes. I pictured some places as depicted by the video.” After surgery, P17 reported being busy and less attentive to video prompts (adherence rate dropped from 100% before surgery to 34% in weeks 1-2 after surgery). Although the frequency of relaxation practice decreased, he reported becoming more adapted to “imagining my own vision while watching videos.” Consistent with P17’s experience, other participants reported continued benefit from the app by gradually grasping relaxation techniques (eg, “learned a lot from the app”), although resumption of regular responsibilities after surgery (eg, work) limited their availability to engage in relaxation practice as before.

### Low-Perceived Benefit Despite High Engagement With The Intervention

#### Adherence Without Perceived Benefit in the Low-Benefit Group

Regarding the low-benefit group’s experience, 2 participants (P6 and P8) showed high adherence to video prompts and short response delays, often demonstrating better adherence than some participants in the high-benefit group. During the first interview before surgery, both P6 and P8 expressed doubt about the value of relaxation exercises. However, because both were retired, they had greater availability and could respond quickly to video prompts. For example, P6 told us: “If I saw there was a notification, I would just open it and play the video, sometimes doing other tasks.” As noted, they typically played the recommended video in the background but were not fully engaged with the content. After surgery, because P6 and P8 did not perceive benefit, they became less compliant with video prompts and mostly chose not to engage with the videos. For the remaining participants who perceived low benefit, although they sometimes complied with video prompts, they did not report a positive experience or develop a routine of practicing relaxation. Interview data indicated that these participants either had difficulty finding time to watch the videos or watched them without full engagement. For example, P18 watched a video before sleep but did not perceive sufficient benefit: “On that day, I just happened to do the breathing exercises when I went to bed. It set the stage for sleep, but certainly not worth that much focus of time.”

Overall, by aligning individual participants’ performance data with their perceptions of the intervention, we found that user engagement with the intervention videos (eg, length of video engagement, delay in attending to a video prompt) may not fully reflect their perceived engagement or ability to derive benefit.

#### Intentional Use as a Key Phenomenon to Differentiate the 2 Groups

As presented above, participants’ performance was largely aligned with perceived benefit from the intervention; however, in some cases, we observed discrepancies between performance and perceptions of the intervention. By triangulating user performance data, self-reported video helpfulness, and interview insights—particularly from participants who showed such discrepancies—we identified intentional use of the intervention content as a key factor in the interview data. We defined “intentional use” as using the intervention content purposefully for desired health-related effects, namely, managing anxiety and pain in our research context. This phenomenon can be considered a qualitative dimension of user engagement that differed between participants who perceived high benefit and those who perceived little benefit.

### Participants Developed Intentional Use of the Intervention (n=7)

Participants in the high-benefit group reported that they would actively engage in relaxation exercises using the MiCarePath app when feeling anxious or in pain, even without prompts. P16, for example, reported experiencing anxiety symptoms for years and being aware of her anxiety patterns. With the intervention, she became more conscious of using it to address her specific patterns of anxiety: “I'm usually already relaxed in the morning, but more at a higher anxiety state in the afternoon. So as long as I had time to watch the videos, I would open the app and practice mindfulness.”

Some participants with little prior experience with relaxation exercises appeared to shift in their engagement with the intervention, from more compliance-based to more intention-driven. For example, P10 reported that she initially responded to prompts to watch relaxation videos before surgery. After surgery, she became more intentional about practicing relaxation to cope with postoperative pain and avoid opioid use: “I was doing it as soon as I got the alerts, but then I learned that I could do them [do meditation exercises] later on. Sometimes it alerted me when I was in the middle of working. This was before my surgery. But now it's like I try to do it at times when I know that I'm stressed, to really get the full effect of it.” P15 gradually developed a habit of intentionally choosing a short relaxation video to address unexpected anxiety episodes, as expressed in the exit interview: “I think, it was going to be the difference between watching or not watching the video, [less about which video to watch]. So sometimes when I felt anxious, I would open the app and pick the shorter one because my goal was just to address my anxiety at that moment.” P19 also reported actively using the intervention; interestingly, she noted that the videos tended to be more helpful when she was more relaxed: “If I do it when I’m more relaxed, I actually pay more attention and the videos will actually help me better more. So I tried to watch them when I felt calm.”

Although some participants perceived little benefit from the intervention, they occasionally reported actively engaging with relaxation exercises without prompts, albeit without developing a clear intention or habit. For example, P8 recalled waiting at a doctor’s appointment and thinking of the intervention. He tried a relaxation exercise during that stressful moment and, for the first time, perceived the video as helpful. He told us, “The (doctor) appointment reminded me of the app so I pulled it up while waiting at the doctor’s place. Other than that, I haven’t experienced [anxiety] a lot, so I could not tell the difference [in my anxiety change before and after watching a video].” Compared with P8, although P5 exhibited low engagement and perceived little benefit from the intervention, she liked the 2-minute diaphragmatic breathing video and actively tried it on 1 occasion when she had time: “A week or 2 before surgery like I said, I was busy preparing like with work and with my house. But I thought about trying the 2-min video (6 breaths per minute), it’s kind of telling you how to do it....But it’s hard to start a habit [practicing relaxation].”

### Participants Applied Relaxation Techniques Without Using the App (n=5)

A subset of participants (n=5) in the high-benefit group learned and remembered scripts of the relaxation exercises and were able to practice meditation without using the app. This may partly explain why these participants, despite subjectively feeling engaged with the intervention, showed lower engagement with relaxation videos (eg, shorter or less frequent use). For example, P10 watched her preferred meditation video multiple times and was able to remember parts of the instructions, which were “replayed” in her mind when she felt anxious. She told us, “I feel like I’ve been able to learn and remember different parts of the video [meditation instructions] even if I did not listen to it. I can still think myself through some of the stuff to relax and to get myself out of a stressful moment. So that’s pretty cool. It’s a skill that I’ve built up over time that I didn’t have at the beginning of all of this [this intervention].” Similarly, P17 preferred the short breathing pacer exercise and actively applied the learned skill over time: “[While practicing deep breathing], you just focus on your breathing, and you don’t think about ‘oh, my heart is racing’. So I could apply this when I felt overwhelmed.”

Moreover, there were situations in which participants intended to watch a video but were unable to interact with the app due to contextual constraints. As a result, some participants applied relaxation techniques without using the app. For example, P11 became capable of proactively using deep breathing techniques when feeling overwhelmed, even when her anxiety was unrelated to surgery, as expressed in the second check-in interview: “I am a contract-based teacher and every time I get a different teaching task. I can be anxious. During the day with my students in the classroom, I couldn’t play the video, so I just reminded myself to take deep breaths when it was overwhelming.”

### Participants With Intentional Use of the Intervention Could Resume Engagement After Experiencing Disruptions

Surgery is considered a major life disruption that requires time, can prompt significant anxiety, and may require additional therapy for recovery. Along with surgery, other contextual disruptions (eg, going on vacation) can lead to temporary or sustained disengagement from mHealth interventions, as reported in prior work [42]. Based on responses from the check-in interviews, several participants in the high-benefit group appeared able to resume engagement after experiencing contextual disruptions to their daily routines. P13 learned to practice deep breathing through the app and was able to intentionally apply the technique when feeling stressed, noting, “I think it benefits me even right now, as I am talking about it. I am slowing down my breathing a little bit.” In week 3 after surgery, she did not use the app at all due to a family trip. Upon returning, she resumed use and achieved a level of video engagement similar to before (127 minutes over the 2 weeks after the trip, compared with 91 minutes before surgery; see Figure 3). By contrast, P2 (low perceived benefit) initially complied with video prompts before surgery but did not find it easy to integrate the meditation exercises into her routines after surgery (eg, “I was not quite engaged,” “Maybe it might help to watch videos right before sleep.”). Following recovery, she reported physical and mental fatigue that disrupted her routine and led to continued disengagement (eg, total watching time was 35 minutes over 4 weeks after surgery).

## Discussion

### Principal Findings

We report data from an exploratory field study of 19 perioperative patients on their experience with and use of a relaxation-based mHealth intervention, MiCarePath, designed to target perioperative anxiety and pain management. Findings indicate 2 distinct user groups: those who perceived high benefit from the intervention (n=11) and those who perceived little to no benefit (n=8). Participants in the high-benefit group remained more engaged with relaxation exercises before and after surgery, showing significant differences in interaction duration with videos (*P*<.001), average response rates (*P*<.001), and rate of perceiving videos as helpful (*P*<.001) compared with participants who reported limited or no benefit. However, for some participants, video engagement did not fully reflect perceived benefit, suggesting a discrepancy between user performance and perceptions of the intervention. Interview data indicated that this discrepancy may be attributed to 2 factors: (1) although participants’ use of the intervention decreased after surgery, they were still able to apply relaxation techniques learned from the videos when needed, even without rewatching them; and (2) participants may have adhered to video prompts without feeling genuinely engaged in the activities, leading to gradual disengagement over time. To characterize this discrepancy, we propose intentional use of the intervention content as an important phenomenon that may distinguish participants who derive high benefit from those who do not. Intentional use can be considered a qualitative dimension of engagement, shifting the focus from the quantity of interaction (eg, amount, frequency, duration) to its quality. This concept complements quantitative measures by capturing whether user interaction with intervention content stems from motivation and intention, thereby distinguishing between passive adherence to intervention delivery and the strategic application of intervention strategies in daily life [53,54]. In this study, participants demonstrating intentional use were able to resume engagement with the intervention after experiencing contextual disruptions to their daily lives.

### Theoretical Implications: Intentional Use and Behavioral Change Frameworks

Our interview data suggest that intentional use of an intervention involves 2 key factors: (1) belief in its ability to alleviate anxiety and pain, and (2) the capacity to schedule and integrate practice into daily routines. These findings align with the Health Action Process Approach (HAPA) [55,56] and the Theory of Planned Behavior (TPB) [57], both of which emphasize intention as central to behavior formation. More specifically, intentional use maps onto key constructs within these frameworks. Participants’ belief in the effectiveness of relaxation techniques parallels outcome expectancies in HAPA and attitudes in TPB, while their ability to apply these techniques within daily routines reflects task self-efficacy in HAPA and perceived behavioral control in TPB. HAPA divides behavior change into 2 stages: the preintentional phase, in which intentions are formed, and the postintentional phase, in which intentions are enacted [55,56]. Within this framework, intention is shaped by task self-efficacy, outcome expectancies, and risk perception [55]. Our study suggests that participants who developed intentional use behaviors may be transitioning into the postintentional phase, actively engaging with the intervention rather than passively receiving content. The identified factor of outcome expectancies reflects participants’ belief in achieving desired outcomes (eg, reduced anxiety or pain), which may explain why some repeatedly watched the same video, driven by anticipated benefits. TPB similarly links intention to attitudes, subjective norms, and perceived behavioral control [57]. Here, attitudes are shaped by the perceived effectiveness of the behavior [57], consistent with participants’ belief in the value of relaxation techniques. Perceived behavioral control extends beyond self-efficacy to include external factors such as time, resources, and environment [58], aligning with participants’ ability to apply relaxation techniques in real-life contexts. Thus, we suggest that intentional use arises when users believe in the effectiveness of the intervention and feel capable of incorporating it into their routines, consistent with facilitators identified in prior work as central to engagement with digital health interventions [54,59].

Furthermore, our findings show that even participants who perceived high benefit may temporarily “disengage” from the intervention, as they may be prescribed additional treatments after surgery. The perioperative context introduces acute stressors—such as surgical anticipation, postoperative pain, and competing clinical demands [60-62]—that can temporarily disrupt observable engagement without necessarily undermining user intention. Our findings suggest that intention may remain stable even when actions on intervention content through the app fluctuate, indicating a decoupling between system-recorded engagement and the underlying behavior change process. This aligns with Yardley’s model, which outlines 4 phases of user engagement with digital behavior change interventions: initial engagement with the intervention only (phase 1), intervention-mediated behavior change (phase 2), sustained behavior change independent of the platform (phase 3), and potential reengagement if needed (phase 4). This model suggests that users may disengage from an intervention platform (eg, an app) while remaining engaged in the behavior change process [63]. Similarly, Perski et al [64] defined engagement as both the extent of usage and a subjective experience, specifically a user’s attention, interest, and affect related to the intervention.

Taken together, user engagement with an intervention—particularly effective engagement—reflects the intervention-mediated behavior change process [63,65]. To better connect the design of mHealth interventions with behavior change theories, designers need to consider the desired type and level of behavior change mediated by the intervention. This perspective aligns with prior agenda-setting work in behavior change and digital health intervention design [66,67]. Such consideration is critical, as the user’s behavior change process may not be entirely driven by the intervention or easily detectable [68]. Furthermore, our research offers a perspective that extends behavioral frameworks by emphasizing contextual disruption as a moderating factor in intention enactment, rather than as a failure of engagement. In this respect, our findings contribute to a more nuanced understanding of user engagement that is sensitive to an individual’s short-term self-care trajectory. Given the variability in users’ motivation, needs, prior skills, and changing contexts, the target level of engagement can be tailored to individual status (eg, existing skills and strategies) and evolving contexts (eg, before and after surgery), rather than defined by uniform expectations of intervention use.

### Design Implications: Supporting Intentional Use in Perioperative mHealth Interventions

#### Overview

In this section, we discuss design implications centered on the concept of intentional use. These implications include (1) accounting for both user performance and perceptions in understanding perioperative patients’ engagement; (2) considering intentional use in the evaluation of mHealth interventions; and (3) designing mHealth interventions to support and shape intentional use.

#### Accounting for Both User Performance and Perceptions in Understanding Perioperative Patients’ Engagement With an Intervention

Assessing user engagement with mHealth interventions requires both objective performance measures and subjective user perceptions, as relying solely on objective metrics may misrepresent engagement by prioritizing frequency over quality [69]. Our study with perioperative patients revealed a potential discrepancy between performance with relaxation videos and perceived benefits. While several low-benefit users consistently followed video prompts with minimal delay, they did not report meaningful benefits. By contrast, intentional use—where users actively incorporate relaxation as a self-management strategy—emerged as a potentially valuable indicator of “effective engagement” and behavior change [63]. This suggests that engagement is not solely driven by system reminders (eg, push notifications), but also by users’ growing recognition of when and how to use the intervention to achieve desired outcomes. As prior research highlights varying definitions and measures of engagement, the concept of “effective engagement”—interactions that lead to meaningful behavioral outcomes [70]—offers a more appropriate lens for evaluation. Given the importance of intentional use in shaping user engagement and behavior change, future mHealth interventions should integrate this factor into system design to enable better personalization and optimization in alignment with behavioral theories.

#### Considering Intentional Use to Evaluate mHealth Interventions

Findings from our study show that participants who benefited most from the app were able to intentionally engage with the intervention content at preferred and needed moments. However, in practice, it remains unclear how to leverage this insight to better understand user experience with mHealth interventions and to optimize intervention algorithms for adaptive interventions. In our study, check-in interviews—through which we learned about participants’ gradual development of intentional use behaviors over time—proved useful. This suggests that periodic assessment of intentional use behavior, as well as progress in developing such behavior, may be valuable for inferring user engagement and determining whether desirable behavioral outcomes (eg, learning a new self-management strategy) are likely to occur.

Indeed, measuring user intention is challenging due to its subjective nature, highlighting the need to develop appropriate measures in future research. Intervention designers may consider using self-report methods to periodically assess intention in using preferred intervention content (eg, new skills, health behaviors) and associated contextual conditions (eg, before sleep, before a doctor’s appointment). One caveat is the potential user burden associated with manual data collection, which may negatively affect overall adherence to the intervention [71]. Furthermore, it remains unclear whether users can accurately express their perceptions and reflect on their intent, particularly when they are in an intermediate stage of intentional use (ie, not fully aware of their intent but gradually developing the inclination to use the intervention) [72]. Future work should continue to explore factors that may influence or indicate intentional use. For example, prior research suggests that engaging with intervention content at a consistent time may indicate higher engagement [73]. In this direction, it would be valuable to explore how to computationally characterize the trajectory of developing intentional use, enabling digital tools to adapt intervention strategies accordingly.

#### Designing to Shape Intentional Use With mHealth Interventions

Our study highlights the potential to shape intentional use of an intervention to achieve behavioral goals. At present, it remains unclear how different system features, such as user onboarding or a feedback dashboard, may support the development of intentional use. Our interview findings suggest that intentional use may involve a belief in the intervention’s ability to produce desired benefits. Accordingly, intervention systems could encourage users to reflect on the benefits they experience [74]. For example, after a relaxation exercise, the app could prompt a simple reflection question: “How do you feel after watching this?” This may help users recognize positive effects over time and reinforce belief in the intervention’s benefits. As intentional use develops—for instance, as users gain a better understanding of when to proactively engage with intervention content—systems could introduce features such as implementation intentions, prompting users to specify actions (eg, engaging with the intervention) and associated conditions (eg, needed moments) [75]. Additionally, interventions could prompt users to reflect on and actively plan future activities based on their detected intentions (eg, [76,77]). For example, a lightweight planning feature might ask, “Would you like to schedule a time for your next relaxation session?” and synchronize with users’ calendars to provide timely nudges [76]. Overall, these design strategies align with behavioral theory by reinforcing self-efficacy and autonomy, rather than promoting compliance with fixed intervention schedules [78].

### Closing Remarks: Practical and Theoretical Implications

#### Theoretical Implications

Intentional, self-initiated use of the intervention—without reliance on system notifications—emerges as a key behavior distinguishing high-benefit users. This finding underscores the theoretical importance of autonomous engagement in mHealth interventions.

#### Practical Implications for the Design and Assessment of mHealth Interventions

Given the potential discrepancies between user performance and perceptions, it is important to consider both aspects when assessing user engagement. When examining user perceptions, priority should be given to self-reported intention, such as perceived behavioral control [79]. Future research should explore the development of assessment methods that effectively capture users’ intentions. When evaluating intervention effectiveness, systems should account for user behaviors that occur outside the system but are reinforced by in-system engagement, to support a more holistic view of users’ progress in the behavior change process. With this perspective, intervention designers should consider incorporating reflective features that promote intentional use or implementation intentions—for example, by prompting users to specify actions and contextual conditions. Looking ahead, future research could investigate gamified meditation exercises to strengthen user intention, alongside visual progress trackers (eg, integrating in-system and off-system activities) to reinforce habit formation.

### Limitations

Although this exploratory study revealed important insights into perioperative patient engagement with a relaxation-based intervention, several limitations should be noted.

First, the study was conducted with a small sample of perioperative patients. As participants were expected to use the intervention around the surgical period, recruitment was subject to strict timing constraints. Despite employing 3 recruitment approaches, we encountered difficulties enrolling participants, primarily due to limited availability and high levels of preoperative stress. These recruitment constraints may have introduced selection bias, as individuals who chose to participate may have been more motivated, more comfortable with technology, or more open to relaxation-based approaches than the broader perioperative population. Nevertheless, we were able to obtain insights from participants with varying levels of intervention engagement, which helped inform refinement of the intervention design and evaluation of its effectiveness.

Second, the study sample was relatively homogeneous, with participants predominantly female and all fluent in English. The predominance of female participants may reflect prior findings that meditation apps tend to be more favored and well-received by females than males [80]. This homogeneity may limit the generalizability of the findings to more diverse populations, including individuals from different cultural backgrounds, non–English-speaking patients, and those with varying levels of health literacy and access to technology. Future work should intentionally recruit more diverse samples to examine how cultural, linguistic, and sociodemographic factors shape engagement with relaxation-based mHealth interventions.

Third, the current intervention does not offer adaptive or personalized tailoring. Findings from this study highlight the importance of facilitating intentional use of interventions. Building on this direction, there is an opportunity to implement a just-in-time adaptive intervention system for perioperative patients and to explore approaches that enhance user proactivity and intentional use over time. Fourth, we conducted multiple check-in interviews during the intervention, which may have been perceived as intrusive and could have influenced user perceptions and experiences. To mitigate this, unless participants asked about a feature in the app, we did not prompt them to engage with the intervention or evaluate their performance. Nevertheless, some participants may have inferred the study’s desired outcomes and, as a result, expressed more favorable views of the intervention during interviews. Accordingly, interview questions were designed to focus on eliciting detailed accounts of participants’ experiences in context. Participants may also have had difficulty recalling details of their engagement with the intervention, leading to potential recall bias. To minimize this, we used a data-prompted interview approach, presenting participants with their anxiety and pain scores, along with screenshots of relaxation videos, to support recall. Finally, several participants dropped out during the postoperative period due to unavailability or recovery, and they may have lacked interest in the intervention; however, we were unable to collect their feedback.

### Conclusions

This exploratory study examined perioperative patients’ engagement with a relaxation-based mHealth intervention (ie, the MiCarePath app) by jointly considering user performance, subjective perceptions, and retrospective perceptions over 3 weeks. Our findings highlight the importance of understanding how patients interpret and apply intervention content within the constraints of the perioperative context. By integrating user engagement data with interview data, this work demonstrates that observable interaction with an intervention may not fully capture perceived value or its role in supporting patients’ self-management during the perioperative period.

A central contribution of this study is the identification of intentional use as a qualitative dimension of engagement that helps explain discrepancies between performance and perceived benefit. Participants who reported greater benefit were not necessarily those with consistently high levels of interaction with intervention content, but rather those who developed the ability to intentionally use relaxation strategies to manage anxiety and pain—including outside the app and after disruptions such as surgery or competing care demands. This finding extends the existing literature on user engagement by emphasizing the quality and purpose of interaction, particularly in short-term, high-burden clinical settings where sustained adherence may be difficult to achieve.

These insights have important implications for the design and evaluation of relaxation-based mHealth interventions in perioperative care. Interventions that support the development of intentional use may better promote nonpharmacological anxiety and pain management, complement clinical care, and reduce reliance on intensive in-person support or medication-based approaches. Future research should explore how intentional use can be fostered and detected over time, as well as how adaptive intervention strategies can be integrated into perioperative care pathways to support scalable, patient-centered outcomes.

## Appendix

Multimedia Appendix 1 The GRAMMS (Good Reporting of a Mixed Methods Study) checklist.

Multimedia Appendix 2 The COREQ (Consolidated Criteria for Reporting Qualitative Research) checklist.

## Abbreviations

COREQ: Consolidated Criteria for Reporting Qualitative Research
GRAMMS: Good Reporting of a Mixed Methods Study
HAPA: Health Action Process Approach
HIPAA: Health Insurance Portability and Accountability Act
mHealth: mobile health
TPB: Theory of Planned Behavior
