# Supplementary material for: Engagement With a Relaxation-Based Mobile Health Intervention for Perioperative Anxiety: Prospective Longitudinal Cohort Mixed Methods Study
Source: JMIR Mhealth Uhealth. 2026 Sep 18;14:e64278. doi: 10.2196/64278 (PMC13633465; doi:10.2196/64278)
Supplement: Supplementary file 1 [file mhealth_v14i1e64278_app1.pdf]

Good Reporting of A Mixed Methods Study (GRAMMS)

Edited by: Xinghui Yan

**Guideline**	**Section: page**
Describe the justification for using a mixed methods approach to the research question	Introduction: p3 (Research Gap), p4 (Objective)
Describe the design in terms of the purpose, priority and sequence of methods	Method: p6 (Study Procedures)
Describe each method in terms of sampling, data collection and analysis	Method: p6 (Study Procedures)<br>Data analysis: p9-10
Describe where integration has occurred, how it has occurred and who has participated in it	Data analysis: p9-10
Describe any limitation of one method associated with the present of the other method	Limitations: p26
Describe any insights gained from mixing or integrating methods	Results: starting from p13, Comparing User Perceptions and Performance Between High- vs Low- Benefit Group

O'Cathain A, Murphy E, Nicholl J. The quality of mixed methods studies in health services research. J Health Serv Res Policy. 2008;13(2):92-98.
